# Comparing SARS-CoV-2 infections in the US Military Health System and national data: opportunities for future pandemic surveillance

**DOI:** 10.3389/fpubh.2025.1714024

**Published:** 2026-01-26

**Authors:** Megan Clare Craig-Kuhn, Laveta Stewart, Erica Sercy, Caryn Stern, Brock Graham, Amber Michel, Edward Parmelee, Stacy Shackelford, Simon Pollett, Timothy Burgess, David R. Tribble

**Affiliations:** 1 Infectious Disease Clinical Research Program, Department of Preventive Medicine and Biostatistics, Uniformed Services University of the Health Sciences, Bethesda, MD, United States; 2 Henry M. Jackson Foundation for the Advancement of Military Medicine, Inc., Bethesda, MD, United States; 3 Joint Trauma System, Joint Base San Antonio, San Antonio, TX, United States

**Keywords:** COVID-19, electronic health records, health systems, SARS-CoV-2, surveillance

## Abstract

**Background:**

The Military Health System offers geographically distributed SARS-CoV-2 incidence estimates to support critical national pandemic surveillance, but this has not been previously assessed. The objective was to identify confirmed, probable, and possible SARS-CoV-2 infections with laboratory and clinical evidence and compare cumulative incidence to the general United States population.

**Methods:**

An observational, retrospective epidemiologic study using medical records from the United States Military Health System (inclusive of active duty) collected from outpatient and inpatient facilities worldwide, both United States Military and non-military treatment facilities. Direct standardization to the general US population was used to calculate sex-adjusted cumulative incidence, stratified by age, for 10 Health and Human Services regions for active duty and non-active duty beneficiary populations, with Spearman’s rho correlations for age and region strata.

**Results:**

Among Military Health System beneficiaries, 2,219,987 cases were identified, with 27.4% laboratory-confirmed cases alongside 35.0% probable and 37.6% possible cases identified using clinical ICD-10-CM evidence. Peaks in cases occurred November 2020–January 2021, August 2021–September 2021, and January 2022. Age-stratified and sex-adjusted cumulative incidence across 10 geographical regions reflected these temporal patterns among both active duty (90% of age and region-specific correlation coefficients >0.7) and non-active duty beneficiaries (80% of age and region-specific correlation coefficients >0.7). Cumulative incidence was higher among active duty beneficiaries compared to the United States general population, particularly those ages 18–49 years, with adjusted cumulative incidence ratios consistently greater than 1. The cumulative incidence ratios for non-active duty beneficiaries were more consistent and closer to 1. The sensitivity analysis of laboratory-confirmed cases among active duty personnel demonstrated consistently lower adjusted cumulative incidence than United States general population.

**Conclusion:**

Temporal patterns in cases among Military Health System beneficiaries reflect cases measured nationally by the Centers for Disease Control and Prevention. Applying a comprehensive algorithm of clinical and laboratory data from a large electronic health system, such as the Military Health System, may improve case capture during an emergent epidemic providing incidence estimates and regional impact in support of U. S. national surveillance.

## Introduction

Accurate identification of severe acute respiratory syndrome coronavirus 2 (SARS-CoV-2) infections is necessary to assess health system burden, and understanding the optimal approach to surveillance in a population is a key ‘lessons learned’ activity for future pandemics. Earlier in the coronavirus disease 2019 (COVID-19) pandemic, polymerase chain reaction (PCR) testing was the gold standard for identifying SARS-CoV-2 infections; however, patients infected with SARS-CoV-2 may not receive a laboratory test, it may not be recorded in the medical record, and home self-testing rapidly expanded. Therefore, a more comprehensive capture of COVID-19 cases has relied on clinical laboratory testing as well as medically attended diagnoses throughout the pandemic. COVID-19 became notifiable to the Centers for Disease Control and Prevention (CDC) in April 2020 with a case report form that includes both laboratory and clinical evidence of infection (1).

The United States (US) Military Health System (MHS) provides healthcare and insurance for a large, diverse population including active duty personnel, retirees, and family members of active duty and retirees. Among the approximately 10 million MHS beneficiaries cumulative from 2020 to 2022 are over one and a half million active duty Service members and National Guard/Reserve members, a population that is younger with a greater proportion of men compared to other MHS beneficiaries and the general US population (2–4).

The Department of Defense implemented surveillance and screening programs that included syndromic surveillance and testing upon entry to training as well as sentinel surveillance including randomly testing 10% of active duty clinical health care personnel and 10% of selected populations living in congregate settings (5). Therefore, it is likely case ascertainment was higher among active duty MHS beneficiaries compared to non-active duty. No similar surveillance programs were implemented on a national level within the US. However, cost-free programs like drive-through testing centers were offered in many locations (6).

Clinical health systems data can be an important tool in public health surveillance and understanding system burden, particularly for a heterogenous population like MHS beneficiaries. With significant differences in exposure risks, surveillance and testing, and demographics, it is unclear how the epidemiology of COVID-19 in active-duty and non-active duty MHS beneficiaries compares to the general US population. Performing this comparison is important for two reasons. Firstly, it allows an assessment of the validity of MHS COVID-19 incidence data inferred by tiered definitions of evidence of infection based on laboratory tests, clinical diagnoses, and syndromic data. Secondly, a notable feature of the early US COVID-19 response were challenges in CDC integration of state and local territory COVID-19 surveillance data (7, 8). Additionally, national consolidation of local COVID-19 data is challenging after expirations of Public Health Emergency declarations (9). Geographically distributed single-payer health system data may contribute significantly to national epidemiological awareness (10), but there has been limited evaluations of how well claims-based data recapitulate national COVID-19 epidemiology.

With these gaps in mind, the first aim of this study is to identify confirmed, probable, and possible cases of SARS-CoV-2 in the MHS applying a customized hierarchy of certainty in evidence of SARS-CoV-2 infection that is uniformly applied across the study period (January 2020 to June 2022). The second aim is to compare the age stratified, sex-adjusted cumulative incidence among MHS beneficiaries to the general US population.

## Methods

### Study design

An observational, retrospective epidemiologic study was conducted among beneficiaries of the MHS.

### Population and setting

All members of the MHS who were active beneficiaries for any amount of time between January 1, 2020 and June 30, 2022 were included in this study. MHS beneficiaries were separated as active duty and non-active duty (i.e., inactive Reserve and National Guard, retirees, dependents, and others). Electronic health systems data relating to care provided in military (direct care) and non-military (purchased care) health facilities in the US and abroad were leveraged for analysis.

### Data

Electronic medical records of MHS beneficiaries are reposed in the Medical Data Repository (MDR) (11). Joint Trauma System Data Custodians identified eligible beneficiaries and de-identified the data before secure transfer to the research team (12). Case numbers among the US general population were from the CDC public use data (downloaded June 6, 2023) (13). US Census data from 2019 provided age and sex stratified populations in each region (downloaded March 30, 2022) (14).

### Definitions

Evidence of SARS-CoV-2 infection was organized in a customized hierarchy of confidence in the case status developed by the study team (Supplementary Table 1). MHS beneficiaries were assessed for evidence of infection sequentially for each level of evidence of infection. First, beneficiaries with a record of positive PCR and/or antigen test(s) were classified as confirmed cases. For those with no positive laboratory test, ICD-10-CM codes for probable cases were assessed next. This process of evaluating evidence of infection was repeated for each descending level of evidence.

The CDC public data case definition included laboratory-confirmed and probable cases (13, 15). Laboratory-confirmed cases required detection of SARS-CoV-2 RNA using molecular amplification. Probable cases in the CDC public data could meet clinical criteria with epidemiologic evidence, presumptive laboratory evidence of antigen or antibody testing indicative of new or recent infection alongside clinical criteria/epidemiologic evidence, or evidence from vital records.

For beneficiaries with evidence of infection on multiple dates within a single tier, the earliest date was used to define infection index date. Region of residence combined Health and Human Services (HHS) regions for those within the 50 US states and the District of Columbia (DC) and World Health Organization regions for all other countries and US territories (16, 17). Hospitalizations for any reason were included.

### Ethical considerations

This research received an exempt determination by the Uniformed Services University of the Health Sciences Human Research Protections Program. A data deidentification process was undertaken before data were made available to researchers, in accordance with applicable Defense Health Agency regulations and agreements.

### Analysis

Descriptive statistics of demographic factors overall in MHS beneficiaries and by active duty status were presented as number and percent without statistical testing and were shown again by case type with chi-square testing. Cumulative incidence within each age/region strata were sex-adjusted through direct standardization, using CDC-reported COVID-19 to identify the number of cases in each stratum and US census data to identify the population of each strata. COVID-19 cases among MHS beneficiaries were also captured in the CDC case counts. Therefore, the number of cases among MHS beneficiaries and the total number of MHS beneficiaries in each region were subtracted from the CDC case data and census numbers, respectively, to create two exclusive populations.

The direct standardization was run separately for active duty and non-active duty MHS beneficiaries in SAS (18). Non-active duty beneficiaries were standardized to the general population including all age categories while only ages 18–64 were assessed among active duty beneficiaries. The publicly available CDC data did not specify whether cases were derived from laboratory and/or clinical evidence. Therefore, our first standardization included both laboratory (i.e., test positivity) and clinical evidence (i.e., ICD-10-CM codes) for cases in the MHS beneficiary population. A sensitivity analysis was conducted by running the direct standardization among only the laboratory-confirmed cases in active duty Service members The standardizations were not conducted in January and February 2020 due to small cell sizes across the strata. Spearman’s correlations were calculated for case types over time and for adjusted cumulative incidence over the study period in each age/region strata for each MHS beneficiary group compared to the general US population. Correlations are categorized as very high (0.9–1.0), high (0.7–0.89), moderate (0.5–0.69), low (0.3–0.49), and negligible (0.0–0.29) (19).

## Results

### Study population

Between January 1, 2020 and June 30, 2022, 10,979,467 beneficiaries were identified comprising 1,889,423 (17.2%) active duty and 9,090,044 (82.8%) non-active duty beneficiaries (Supplementary Table 2). These numbers represent cumulative beneficiaries over the study period, meaning they were not all on active duty at the same time or active beneficiaries at the same time. Active duty beneficiaries were mostly younger adults (ages 18–49) (97.7%) and male (82.1%) while non-active duty beneficiaries had a broader age distribution and a higher percent were females (54.3%). The largest proportion of both active duty (24.2%) and non-active duty (28.2%) beneficiaries lived in HHS Region 4 (AL, FL, GA, KY, MS, NC, SC, TN). For active duty beneficiaries this was following by 16.9% in Region 9 (AZ, CA, HI, NV) and 13.8% in Region 6 (AR, LA, NM, OK, TX). For non-active duty beneficiaries this was followed by 15.1% in Region 6 (AR, LA, NM, OK, TX) and 12.9% in Region 3 (DE, DC, MD, PA, VA, WV). Most (68.3%) non-active duty beneficiaries were dependents of active duty Service members, 26.0% were retirees, 4.4% inactive National Guard/Reserve members, and 1.4% other.

### Case identification and trends

A total 2,219,987 COVID-19 cases were identified. Confirmed cases represented 27.4% of cases, probable included 35.0%, and possible included 37.6% (with 35.3% being acute possible infections and 2.3% being non-acute possible cases; Supplementary Table 3). Among confirmed cases, 61.9% were active duty service members, 65.2% male and 77.6% between the ages of 18–49 years. Hospitalization frequency in the 30 days after the index date ranged from 1.7% among confirmed cases to 17.0% in acute possible cases. Hospitalization in the 14 days before index date (range 0.2–3.1%) and days 31–45 after index date (range 0.2–0.4%) were less common. While 0.42% of active duty Service members were hospitalized in the 30 days following index date, this occurred in 2.41% of non-active duty beneficiaries (highest in those aged 50–64 at 2.0% and 65 + at 6.2%) (not shown in table).

**Figure 1:**
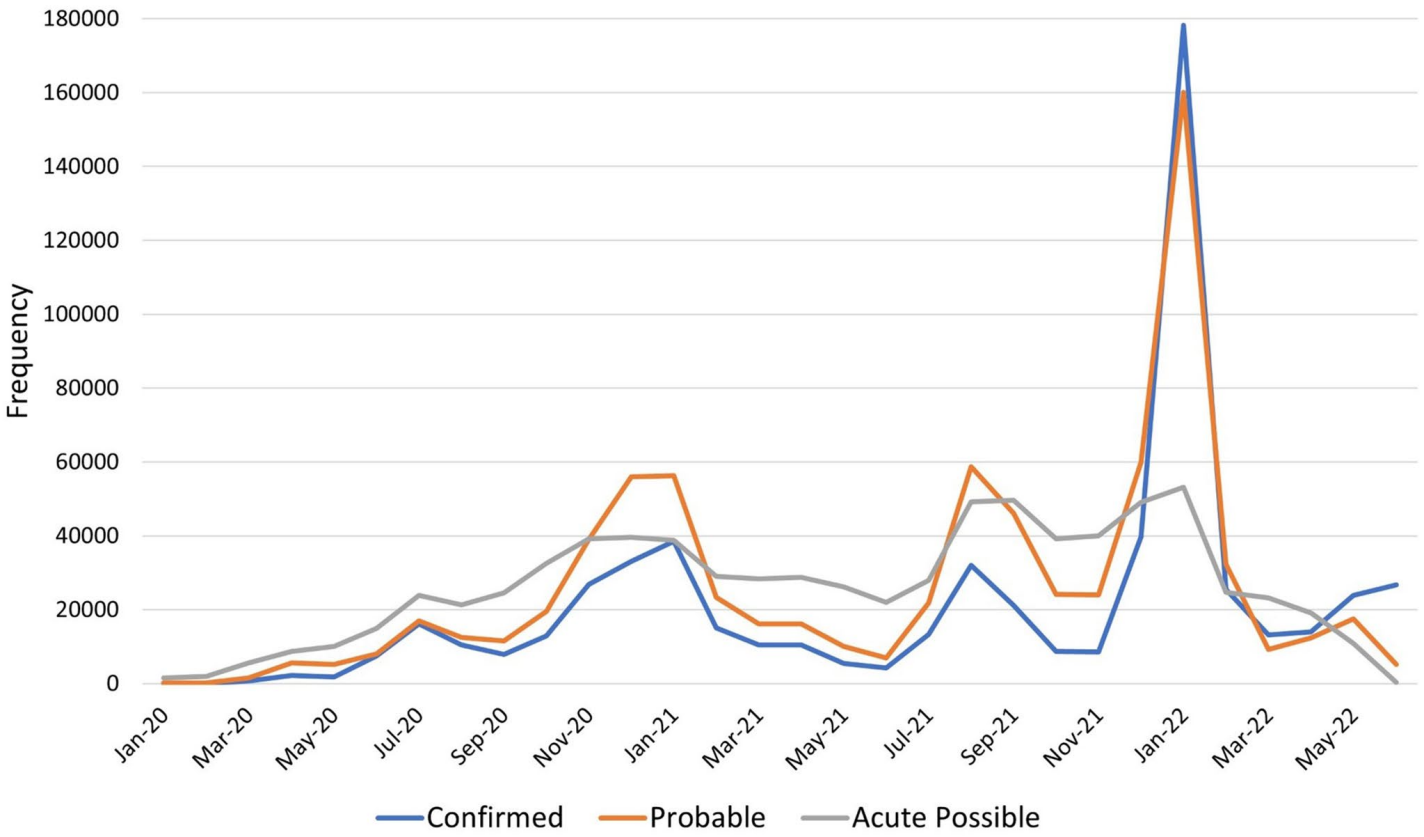
Frequency of confirmed, probable, and possible SARS-CoV-2 infections by month, January 2020 to June 2022. The blue line indicates the number of confirmed SARS-CoV-2 infections by month, orange probable cases, and gray acute possible cases. Non-acute possible cases (those with evidence of history of COVID-19 but no evidence on the acute infection) are not shown on this graph due to low numbers.

Confirmed, probable, and acute possible cases showed peaks in cases November 2020–January 2021, August 2021–September 2021, and January 2022 (Figure 1). Correlation of the case groups over time ranged from very high between confirmed and probable (0.94, *p* < 0.0001), to high between probable and acute possible (0.73, p < 0.0001), and moderate between confirmed and possible (0.50, *p* = 0.0072; Supplementary Table 4). Numbers of non-acute possible cases remained very low (average monthly frequency 2,711) but did increase across this time period, with a small peak January–February 2022 (not shown in figure).

### Comparison of COVID-19 in MHS and general US populations

To conduct the age stratified, sex-adjusted cumulative incidence standardization, 437,752 (4.0%) MHS beneficiaries (including 95,382 cases) were excluded entirely due to missing age, sex, or region, or if they were located outside the 50 US states and DC, or if they were active duty and aged 17 or 65 or older.

**Figure 2:**
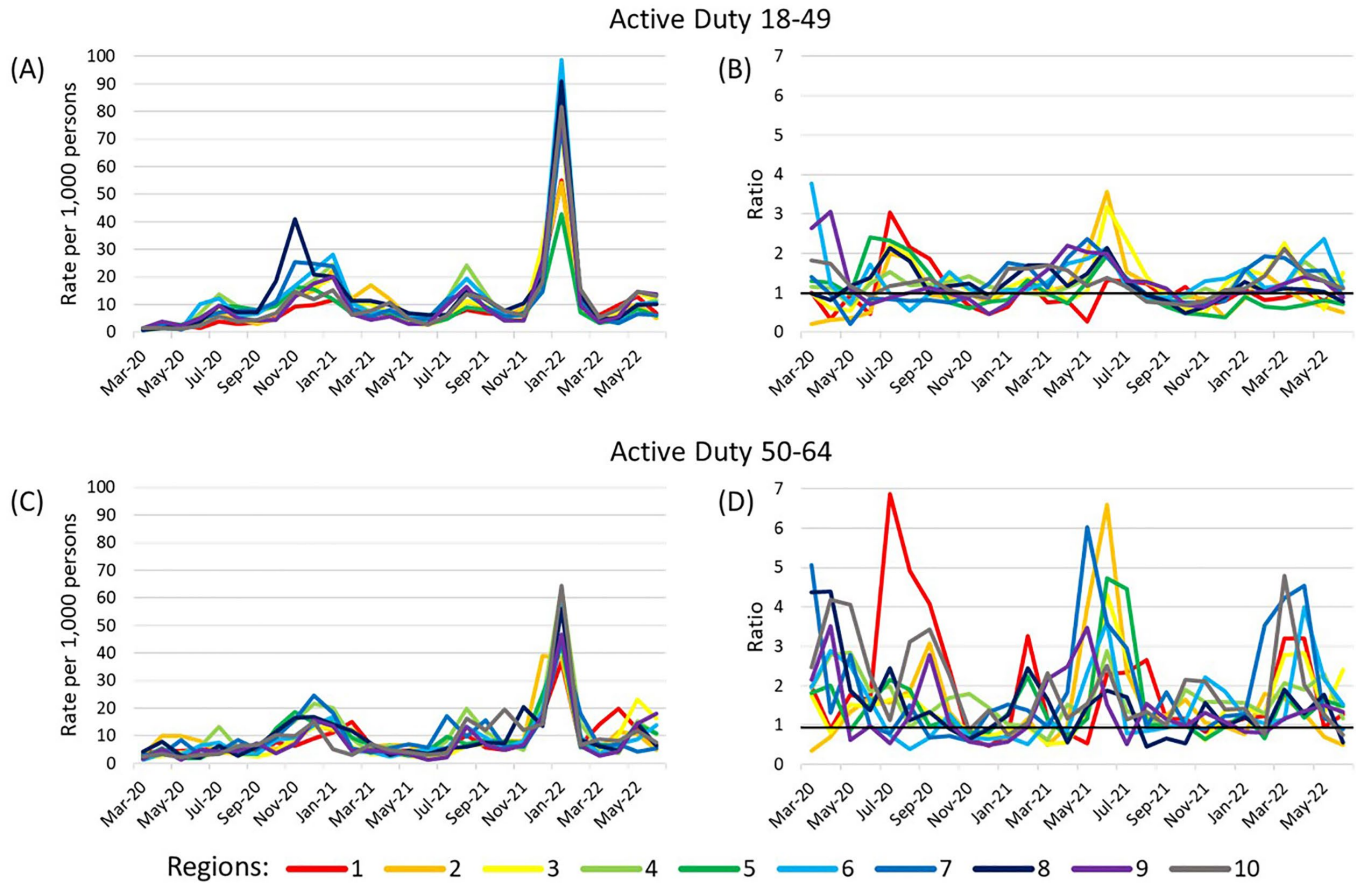
Adjusted cumulative incidence of SARS-CoV-2 among active duty beneficiaries and ratios compared to US population. **(A,B)** show results for active duty beneficiaries ages 18–49 years and **(C,D)** show active duty beneficiaries ages 50–64 years. Cumulative incidence (per 1,000 persons) was adjusted for sex (male or female) and stratified by age (18–49, 50–64) within each region. The reference population is the general US population within each Health and Human Services region with the number of MHS beneficiaries removed from the total region population and number of cases among MHS beneficiaries removed from the number of US cases in the region. Ratios compare adjusted cumulative incidence of SARS-CoV-2 among the MHS population to the general US population within each region/age strata. A ratio >1 indicates the adjusted cumulative incidence is higher in the MHS population while a ratio <1 indicates the adjusted cumulative incidence is lower among the MHS. A dark horizontal line on the ratio graphs at 1 indicate where the rates of the two populations were equal. Region 1 (CT, ME, MA, NH, RI, VT); Region 2 (NJ, NY); Region 3 (DE, DC, MD, PA, VA, WV); Region 4 (AL, FL, GA, KY, MS, NC, SC, TN); Region 5 (IL, IN, MI, MN, OH, WI); Region 6 (AR, LA, NM, OK, TX); Region 7 (IA, KS, MO, NE); Region 8 (CO, MT, ND, SD, UT, WY); Region 9 (AZ, CA, HI, NV); Region 10 (AK, ID, OR, WA).

The adjusted cumulative incidence of COVID-19 among active duty beneficiaries had peaks November 2020–January 2021, August 2021–September 2021, and January 2022 (Figure 2). However, among the 50–64 year olds, there was some variation in which month each region had a peak in cases. The ratios of adjusted cumulative incidence between MHS beneficiaries and the general US population varied greatly among 50–64 year olds, with many being greater than 1. Correlations of adjusted cumulative incidence between MHS beneficiaries and the general US population over time for this age group were moderate for Regions 1 (0.64, *p* = 0.0003) and 7 (0.62, *p* = 0.0004), very high for Region 4 (0.92, *p* < 0.0001), and high for all others (range 0.75–0.87; Supplementary Table 5). There was a smaller range of adjusted cumulative incidence ratios among the 18–49 year olds and many were greater than 1. Correlations were very high for Regions 4, 6, 9, and 10, and high for all others (range 0.75–0.98).

**Figure 3:**
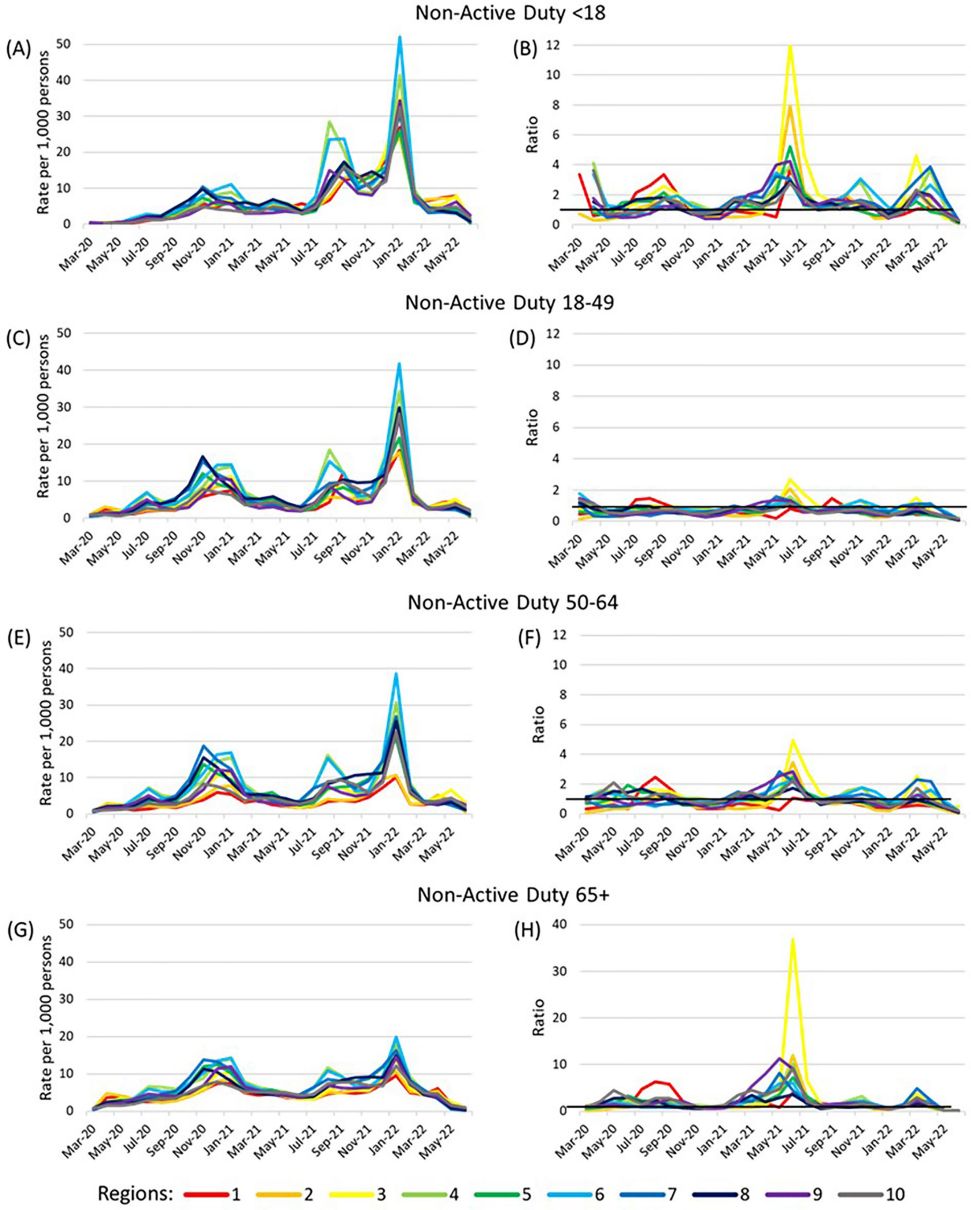
Adjusted cumulative incidence of SARS-CoV-2 among non-active duty beneficiaries and ratios compared to US population. **(A,B)** show results for non-active duty beneficiaries under 18 years, **(C,D)** non-active duty beneficiaries ages 18–49 years, **(E,F)** non-active duty beneficiaries ages 50–64 years, and **(G,H)** non-active duty beneficiaries ages 65 years and older. Cumulative incidence (per 1,000 persons) was adjusted for sex (male or female) and stratified by age (<18, 18–49, 50–64, 65+) within each region. The reference population is the general US population within each Health and Human Services region with the number of MHS beneficiaries removed from the total region population and number of cases among MHS beneficiaries removed from the number of US cases in the region. A dark horizontal line on the ratio graphs at 1 indicate where the rates of the two populations were equal. Region 1 (CT, ME, MA, NH, RI, VT); Region 2 (NJ, NY); Region 3 (DE, DC, MD, PA, VA, WV); Region 4 (AL, FL, GA, KY, MS, NC, SC, TN); Region 5 (IL, IN, MI, MN, OH, WI); Region 6 (AR, LA, NM, OK, TX); Region 7 (IA, KS, MO, NE); Region 8 (CO, MT, ND, SD, UT, WY); Region 9 (AZ, CA, HI, NV); Region 10 (AK, ID, OR, WA).

Among non-active duty beneficiaries, the peak in adjusted cumulative incidence from November 2020–January 2021 was smallest among the youngest age group (<18) and the January 2022 peak was smallest among the oldest age group (65+) (Figure 3). The adjusted cumulative incidence ratios for the 18–49 year olds and the 50–64 year olds were typically close to 1, while the ratios among those under 18 and those 65 + were more frequently greater than 1. The correlations of adjusted cumulative incidence over time between non-active duty MHS beneficiaries and the general US population were high or very high in all regions for those under 18 years old (range 0.75–0.92) and ages 18–49 years (range 0.71–0.91; Supplementary Table 5). Among 50–64 year olds, the correlation for Region 2 was moderate (0.69, *p* < 0.0001) while other regions had high or very high correlation (range 0.79–0.90). Among those ages 65 years and older, the correlation was low in Region 2 (0.48, *p* = 0.01), moderate for Regions 1, 3, 5, 8, 9, and 10 (range 0.60–0.68), and high in Regions 4, 6, and 7 (range 0.72–0.77).

### Sensitivity analysis

**Figure 4:**
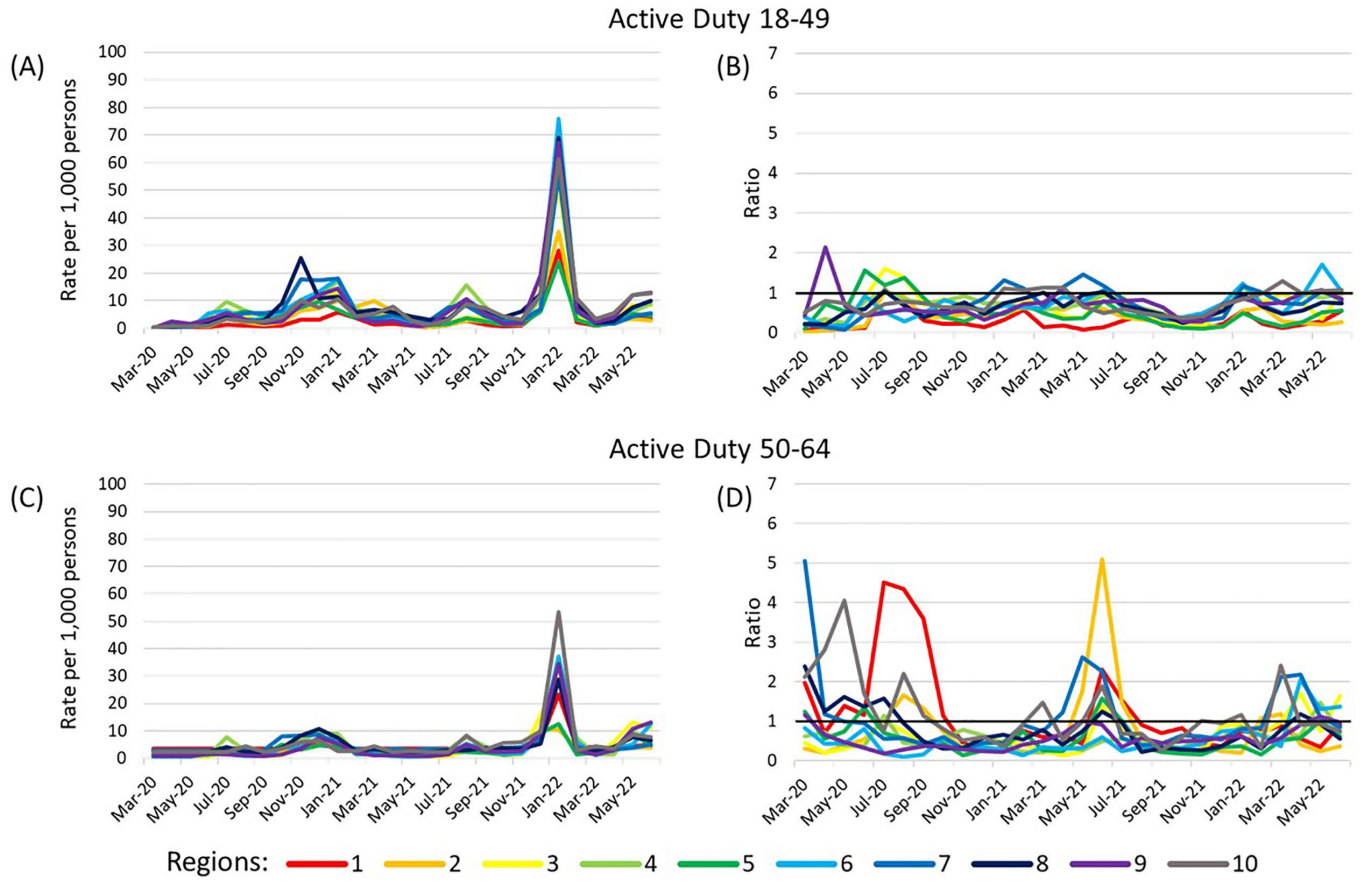
Adjusted cumulative incidence of laboratory-confirmed SARS-CoV-2 among active duty beneficiaries and ratios to US population. **(A,B)** show results for active duty beneficiaries ages 18–49 years and **(C,D)** show active duty beneficiaries ages 50–64 years. Cumulative incidence (per 1,000 persons) was adjusted for sex (male or female) and stratified by age (18–49, 50–64) within each region. The reference population is the general US population within each Health and Human Services region with the number of MHS beneficiaries removed from the total region population and number of laboratory-confirmed cases among MHS beneficiaries removed from the number of US cases in the region. A dark horizontal line on the ratio graphs at 1 indicate where the rates of the two populations were equal. Region 1 (CT, ME, MA, NH, RI, VT); Region 2 (NJ, NY); Region 3 (DE, DC, MD, PA, VA, WV); Region 4 (AL, FL, GA, KY, MS, NC, SC, TN); Region 5 (IL, IN, MI, MN, OH, WI); Region 6 (AR, LA, NM, OK, TX); Region 7 (IA, KS, MO, NE); Region 8 (CO, MT, ND, SD, UT, WY); Region 9 (AZ, CA, HI, NV); Region 10 (AK, ID, OR, WA).

The sensitivity analysis restricting to only laboratory-confirmed COVID-19 in active duty Service members demonstrated consistently lower adjusted cumulative incidence compared to the original analysis, especially for 50–64 year olds (Figure 4). The adjusted cumulative incidence ratios among 18–49 year olds were within a small range, while 50–64 year olds had more variation, but ratios were frequently lower than 1 for both age groups. The correlation of adjusted cumulative incidence over time between active duty Service members and the general US population among 18–49 year old decreased in all 10 regions compared to the analysis with all case types (range 0.60–0.96; Supplementary Table 5). Among 50–64 year olds, the change in correlation values was not consistent; correlation was moderate in Regions 1 (0.67, *p* < 0.0001) and 2 (0.53, *p* = 0.0039), very high in Regions 4 (0.92, *p* < 0.0001), and 9 (0.93, *p* < 0.0001), and high in all others (range 0.71–0.80).

### Laboratory testing and exposure codes

PCR tests were recorded more frequently than antigen tests across the study period (Supplementary Figure 1). The percent positive for both test types increased during the peaks in cases seen in November 2020–January 2021, August 2021–September 2021, and January 2022. The percent positive for PCR tests is high in the first several months of 2020 but few tests were recorded.

ICD-10-CM code Z20.822 was used most frequently before January 1, 2021, after which point the COVID-19 specific code Z20.828 was used more frequently (Supplementary Figure 2). The codes Z20.89 and Z20.9 typically each had fewer than 2,000 recorded per month and never more than 5,000 per month during the study period and were therefore excluded from the case definitions.

## Discussion

These results provide key estimates of time-varying COVID-19 rates across the entire MHS and serve as an evaluation of optimal pandemic surveillance approaches in the MHS. We noted MHS surveillance data recapitulated national US COVID-19 epidemiological trends and should be considered as a complementary source of data to support national pandemic response in future public health emergencies.

There are several factors to consider when comparing active duty beneficiaries to non-active duty and the US general population. While active duty Service members have an increased risk of infection due to congregate living and requirement to perform duties in-person in close proximity to others (20), the sampling programs implemented by the Department of Defense among active duty Service members likely resulted in differential case ascertainment (5). Surveillance screening programs would be expected to ascertain asymptomatic or minimally symptomatic cases that would otherwise not have been identified. Additionally, the results of the sampling programs are expected to have been recorded in the MHS electronic health records. Laboratory-confirmed cases demographically resembled the active duty Service member population (mostly 18–49 years old and male), presumably as a result of the testing strategies implemented among this group (5).

Considering the differences in testing and data collection systems between the MHS and CDC, the measure of incidence among active duty beneficiaries most comparable to that obtained for the US general population is likely between the two estimates presented for active duty here. While the lower hospitalization among active duty beneficiaries was largely due to higher rates among older adults (with there being few older adults on active duty), there was very high vaccination coverage among the active-duty population, which reduces severe disease (21, 22).

Probable and possible cases utilized ICD-10-CM codes which require interaction with the healthcare system for the codes to be recorded. Multiple studies have demonstrated the validity and utility of using ICD-10-CM codes for identifying COVID-19, including most of the codes included in our case definitions (23–27). Over time during the pandemic, PCR testing became widely available in venues outside the military health system facilities and home antigen tests were widely accessible (6). Patients could be tested externally but seek care internally, in which case the laboratory results may not be recorded. Groups with more severe symptoms and outcomes could be more likely to seek treatment and therefore would be more likely to have diagnoses recorded in the health system, while asymptomatic or mildly symptomatic would be less likely to be identified. This could be the reason for the higher proportion of hospitalizations among possible and probable cases, though it is unknown why no laboratory tests were recorded in hospitalized cases. Relying on laboratory data alone can miss cases who lacked laboratory results within MDR records.

The number of cases identified over time in the MHS mirrors patterns identified with national CDC data (28, 29). The correlation of adjusted cumulative incidence over time between MHS beneficiary groups and the general US population was high or very high for 83.3% of region/age strata, while only 15% of strata had moderate correlation, and a single strata had low correlation. These patterns include moderate peaks November 2020–January 2021 (national data 7-day average reaching above 250,000 cases) and August 2021–September 2021 (national data 7-day average reaching above 160,000 cases) (29), the latter being predominated by the Delta variant (30). Both the MHS and CDC data captured a peak of large magnitude in January 2022 (national data 7-day average reaching above 800,000 cases) which was predominated by the Omicron variant (29, 30). This consistency is reassuring that these two systems captured cases similarly in the environment of changing policy, testing capability, and vaccination uptake, and despite known differences in the demographic and geographic distribution of the MHS and the general US populations.

Similar peaks in case numbers were also seen in the stratified analyses among active duty and non-active duty MHS beneficiaries. However, we noted spatial heterogeneity in the magnitude and timing of epidemic peaks, which may have been partly explained by small differences in the timing of variant predominance from state-to-state, heterogeneity in population vaccination and immunity, and other drivers of geographic viral dispersal (e.g., population structure and connectivity) (30, 31).

A limitation of the study is possible misclassification of infections using ICD-10-CM evidence, as some of these cases may be true negatives. Second, testing programs among active duty personnel could lead to differential case ascertainment. This is one reason the MHS beneficiary population was separated for analysis. Additionally, the last 2 months of data from the MHS may have experienced some lag in reporting and therefore may be undercounting cases in those months. Finally, the data were less reliable during the first several months of 2020 for both the MHS and CDC data. Case definitions, testing, and documentation had no consensus at that time and case numbers were low.

Future assessment of MHS data in the post-pandemic period will be necessary to address optimal ICD-10-CM codes used to define probable and possible cases. Due to ongoing changes in testing and surveillance, and return of other acute respiratory infections, the ICD-10-CM used in case definitions may need to be adjusted to better differentiate SARS-CoV-2 from other respiratory pathogens and best complement laboratory testing data.

Our results highlight future improvements in MHS case definitions and documentation in future pandemics, with consensus enabled by early comparisons of MHS and CDC national data following the methodological approaches demonstrated here. These demographically stratified estimates of COVID-19 incidence provide important epidemiological parameters which may support predictions, resource planning, and countermeasure implementation for future pandemics.

## Data Availability

The data analyzed in this study is subject to the following licenses/restrictions: The dataset that support the findings of this study are available from the Military Health System Medical Data Repository, but restrictions apply to the availability of these data, which were used under license for the current study, and so are not publicly available. Requests to access these datasets should be directed to dha.ncr.pcl.mbx.data-sharing@health.mil.
