# Supplementary material for: Comparing SARS-CoV-2 infections in the US Military Health System and national data: opportunities for future pandemic surveillance
Source: Front Public Health. 2026 Jan 26;13:1714024. doi: 10.3389/fpubh.2025.1714024 (PMC12883756; doi:10.3389/fpubh.2025.1714024)
Supplement: Supplementary file 1 [file Table_1.docx]

Supplementary Table 1. Hierarchy of evidence of SARS-CoV-2 infection.

| **ICD-10-CM** | **Definition** | **Requirements** |
| --- | --- | --- |
| **Confirmed Cases** | | |
| n/a | Positive laboratory results from molecular and/or antigen test. Positive tests are taken as cases even when negative tests are recorded within a short time frame. | - Earliest positive test in study period |
| **Probable Cases** | | |
| U07.1 | 2019-nCoV acute respiratory disease, COVID-19, virus identified | - Earliest occurrence of any one of these codes in study period |
| U07.2 | COVID-19, virus not identified (clinically diagnosed) (*this code is not used in the US, but is used internationally*) |  |
| J12.82 | Pneumonia due to coronavirus disease 2019 |  |
| J12.81 | Pneumonia due to SARS-associated coronavirus |  |
| B34.2 | Coronavirus, unspecified |  |
| B97.2 | Coronavirus as the cause of disease classified elsewhere |  |
| **Possible Cases** | | |
| **Code Z20.822 and/or Z20.828 indicating contact with and (suspected) exposure to a SARS-CoV-2 infected person AND any one of the following conditions within ±14 days:** | | |
| J12.89 | Other viral pneumonia | - Earliest occurrence of any one of these codes in study period - Must be within ±14 days of suspected exposure |
| J12.9 | Viral pneumonia unspecified |  |
| J16.8 | Pneumonia due to other specified infectious organism |  |
| J17 | Pneumonia in diseases classified elsewhere |  |
| J18.0 | Bronchopneumonia, unspecified organism |  |
| J18.1 | Lobar pneumonia, unspecified organism |  |
| J18.2 | Hypostatic pneumonia, unspecified organism |  |
| J18.8 | Other pneumonia, unspecified organism |  |
| J18.9 | Pneumonia, unspecified organism |  |
| J84.111 | Idiopathic interstitial pneumonia not otherwise specified |  |
| J80 | Acute Respiratory Distress Syndrome |  |
| J06.0 | Acute laryngopharyngitis |  |
| J06.9 | Acute upper respiratory infection, unspecified |  |
| J20.8 | Acute bronchitis due to other specified organisms |  |
| J20.9 | Acute bronchitis, unspecified |  |
| J22 | Unspecified acute lower respiratory infection |  |
| J40 | Bronchitis, not specified as acute or chronic |  |
| J96.0 | Acute respiratory failure |  |
| J98.8 | Other specified respiratory disorders |  |
| M35.81 | Multisystem inflammatory syndrome |  |
| R06.03 | Acute Respiratory Distress |  |
| R05 | Cough |  |
| R06.02 | Shortness of Breath |  |
| R06.00 | Dyspnea, unspecified |  |
| R06.09 | Other forms of dyspnea |  |
| R43.0 | Anosmia |  |
| R43.2 | Ageusia |  |
| R43.8 | Other disturbances of smell and taste |  |
| R43.9 | Unspecified disturbances of smell and taste |  |
| R41.0 | Disorientation, unspecified |  |
| R41.82 | Altered mental status, unspecified |  |
| R41.9 | Unspecified symptoms and signs involving cognitive functions and awareness |  |
| R07.89 | Other chest pain |  |
| R07.9 | Chest pain, unspecified |  |
| **Code Z20.822 and/or Z20.828 indicating contact with and (suspected) exposure to a SARS-CoV-2 infected person AND any two of the following conditions, all within ±14 days of each other:** | | |
| R50.9 | Fever, unspecified | - Earliest occurrence of two of these codes within ±14 days of each other in study period - Must also be within ±14 days of suspected exposure |
| R68.83 | Chills (without fever) |  |
| M79.1 | Myalgia |  |
| R51 | Headache |  |
| R07.0 | Pain in throat |  |
| R11.0 | Nausea |  |
| R11.10 | Vomiting unspecified |  |
| R11.11 | Vomiting without nausea |  |
| R11.2 | Nausea with vomiting, unspecified |  |
| R19.7 | Diarrhea, unspecified |  |
| R53.83 | Other fatigue |  |
| R09.81 | Nasal congestion |  |
| **Non-acute possible cases** | | |
| U09.9 | Post COVID-19 condition, unspecified | - Earliest occurrence of either code in study period |
| Z86.16 | Personal history of COVID-19 |  |
