# Supplementary material for: Comparing SARS-CoV-2 infections in the US Military Health System and national data: opportunities for future pandemic surveillance
Source: Front Public Health. 2026 Jan 26;13:1714024. doi: 10.3389/fpubh.2025.1714024 (PMC12883756; doi:10.3389/fpubh.2025.1714024)
Supplement: Supplementary file 2 [file Table_2.docx]

Supplementary Table 2. MHS beneficiary demographics overall and by active duty status.

|  |  | | Active Duty Status | | | |
| --- | --- | --- | --- | --- | --- | --- |
|  | All MHS Beneficiaries  N=10,979,467 | | Active Duty^1^  N=1,889,423 | | Non-active Duty  N=9,090,044 | |
|  | N | % | N | % | N | % |
| Age |  |  |  |  |  |  |
| 0-17 | 2,164,998 | 19.7 | 2,480^2^ | 0.1 | 2,162,518 | 23.8 |
| 18-49 | 4,659,873 | 42.4 | 1,845,632 | 97.7 | 2,814,241 | 31.0 |
| 50-64 | 1,573,722 | 14.3 | 41,230 | 2.2 | 1,532,492 | 16.9 |
| 65+ | 2,580,364 | 23.5 | 81 | 0.0 | 2,580,283 | 28.4 |
| Unknown/Missing | 510 | 0.0 | 0 | 0.0 | 510 | 0.0 |
| Sex |  |  |  |  |  |  |
| Male | 5,703,647 | 52.0 | 1,551,944 | 82.1 | 4,151,703 | 45.7 |
| Female | 5,275,685 | 48.1 | 337,471 | 17.9 | 4,938,214 | 54.3 |
| Unknown/Missing | 135 | 0.0 | 8 | 0.0 | 127 | 0.0 |
| Region of Residence |  |  |  |  |  |  |
| International—WHO regions |  |  |  |  |  |  |
| African Region | 1,698 | 0.0 | 614 | 0.0 | 1,084 | 0.0 |
| Region of the Americas (excluding US) | 148,740 | 1.4 | 4,500 | 0.2 | 144,240 | 1.6 |
| South-East Asian Region | 1,720 | 0.0 | 172 | 0.0 | 1,548 | 0.0 |
| European Region | 97,898 | 0.9 | 66,574 | 3.5 | 31,324 | 0.3 |
| Eastern Mediterranean Region | 6,847 | 0.1 | 5,532 | 0.3 | 1,315 | 0.0 |
| Western Pacific Region | 102,932 | 0.9 | 75,447 | 4.0 | 27,485 | 0.3 |
| US Domestic—HHS regions |  |  |  |  |  |  |
| Region 1 (CT, ME, MA, NH, RI, VT) | 272,832 | 2.5 | 32,941 | 1.7 | 239,891 | 2.6 |
| Region 2 (NJ, NY) | 319,055 | 2.9 | 55,884 | 3.0 | 263,171 | 2.9 |
| Region 3 (DE, DC, MD, PA, VA, WV) | 1,410,063 | 12.8 | 239,234 | 12.7 | 1,170,829 | 12.9 |
| Region 4 (AL, FL, GA, KY, MS, NC, SC, TN) | 3,024,720 | 27.6 | 457,423 | 24.2 | 2,567,297 | 28.2 |
| Region 5 (IL, IN, MI, MN, OH, WI) | 818,301 | 7.5 | 85,496 | 4.5 | 732,805 | 8.1 |
| Region 6 (AR, LA, NM, OK, TX) | 1,634,181 | 14.9 | 260,600 | 13.8 | 1,373,581 | 15.1 |
| Region 7 (IA, KS, MO, NE) | 457,050 | 4.2 | 67,739 | 3.6 | 389,311 | 4.3 |
| Region 8 (CO, MT, ND, SD, UT, WY) | 534,341 | 4.9 | 87,875 | 4.7 | 446,466 | 4.9 |
| Region 9 (AZ, CA, HI, NV) | 1,438,698 | 13.1 | 318,335 | 16.9 | 1,120,363 | 12.3 |
| Region 10 (AK, ID, OR, WA) | 635,678 | 5.8 | 117,893 | 6.2 | 517,685 | 5.7 |
| Unknown/Missing | 74,813 | 0.7 | 13,164 | 0.7 | 61,649 | 0.7 |
| Race |  |  |  |  |  |  |
| American Indian or Alaska Native | 45,186 | 0.4 | 19,386 | 1.0 | 25,800 | 0.3 |
| Asian or Pacific Islander | 272,112 | 2.5 | 114,014 | 6.0 | 158,098 | 1.7 |
| Black or African American | 828,215 | 7.5 | 317,124 | 16.8 | 511,091 | 5.6 |
| White | 3,451,214 | 31.4 | 1,314,364 | 69.6 | 2,136,850 | 23.5 |
| Other | 204,318 | 1.9 | 99,159 | 5.3 | 105,159 | 1.2 |
| Unknown/Missing | 6,178,422 | 56.3 | 25,376 | 1.3 | 6,153,046 | 67.7 |
| Hispanic Ethnicity |  |  |  |  |  |  |
| Yes | 521,339 | 4.8 | 325,581 | 17.2 | 195,758 | 2.2 |
| No | 4,278,160 | 39.0 | 1,540,594 | 81.5 | 2,737,566 | 30.1 |
| Unknown/Missing | 6,179,968 | 56.3 | 23,248 | 1.2 | 6,156,720 | 67.7 |
| MHS Beneficiary Status |  |  |  |  |  |  |
| Active duty^1^ | 1,889,423 | 17.2 | 1,889,423 | 100.0 | 0 | 0.0 |
| Inactive Nat. Guard/Reserve | 398,318 | 3.6 | 0 | 0.0 | 398,318 | 4.4 |
| Retirees | 2,360,037 | 21.5 | 0 | 0.0 | 2,360,037 | 26.0 |
| Dependents | 6,206,215 | 56.5 | 0 | 0.0 | 6,206,215 | 68.3 |
| Other | 124,612 | 1.1 | 0 | 0.0 | 124,612 | 1.4 |
| Unknown | 862 | 0.0 | 0 | 0.0 | 862 | 0.0 |
| Member |  |  |  |  |  |  |
| Self | 4,717,604 | 43.0 | 1,889,423 | 100.0 | 2,828,181 | 31.1 |
| Spouse | 3,390,348 | 30.9 | 0 | 0.0 | 3,390,348 | 37.3 |
| Child/Ward | 2,853,528 | 26.0 | 0 | 0.0 | 2,853,528 | 31.4 |
| Parent | 17,986 | 0.2 | 0 | 0.0 | 17,986 | 0.2 |
| Branch of Service^3^ |  |  |  |  |  |  |
| Air Force | 2,878,417 | 26.2 | 438,480 | 23.2 | 2,439,937 | 26.8 |
| Army | 4,694,753 | 42.8 | 733,119 | 38.8 | 3,961,634 | 43.6 |
| Coast Guard | 243,516 | 2.2 | 46,123 | 2.4 | 197,393 | 2.2 |
| Marine Corps | 862,039 | 7.9 | 245,559 | 13.0 | 616,480 | 6.8 |
| Navy | 2,217,253 | 20.2 | 414,069 | 21.9 | 1,803,184 | 19.8 |
| Other | 34,760 | 0.3 | 359 | 0.0 | 34,401 | 0.4 |
| Unknown | 48,729 | 0.4 | 11,714 | 0.6 | 37,015 | 0.4 |
| Rank^3^ |  |  |  |  |  |  |
| Enlisted E1-E3 | 794,281 | 7.2 | 480,637 | 25.4 | 313,644 | 3.5 |
| Enlisted E4-E6 | 4,354,676 | 39.7 | 908,425 | 48.1 | 3,446,251 | 37.9 |
| Enlisted E7-E9 | 3,223,965 | 29.4 | 183,579 | 9.7 | 3,040,386 | 33.5 |
| Officer O1-O3 | 586,357 | 5.3 | 170,716 | 9.0 | 415,641 | 4.6 |
| Officer O4-O6 | 1,688,988 | 15.4 | 117,580 | 6.2 | 1,571,418 | 17.3 |
| Officer O7-O10 | 25,519 | 0.2 | 1,195 | 0.1 | 24,324 | 0.3 |
| Warrant Officer W1-W5 | 303,963 | 2.8 | 27,197 | 1.4 | 276,766 | 3.0 |
| Unknown | 1,718 | 0.0 | 104 | 0.0 | 1,614 | 0.0 |

^1^Active duty includes Reserve and National Guard personnel on active duty status.

^2^Active duty in the 0-17 age group are all 17 years old.

^3^For non-active duty beneficiaries, branch and rank refer to former branch and rank for retirees and branch and rank of sponsoring service member for dependents.

Abbreviations: MHS, military health system; WHO, World Health Organization; HHS, Health and Human Services; states use postal service abbreviations.
