# Supplementary material for: Comparing SARS-CoV-2 infections in the US Military Health System and national data: opportunities for future pandemic surveillance
Source: Front Public Health. 2026 Jan 26;13:1714024. doi: 10.3389/fpubh.2025.1714024 (PMC12883756; doi:10.3389/fpubh.2025.1714024)
Supplement: Supplementary file 3 [file Table_3.docx]

Supplementary Table 3. MHS beneficiary characteristics by evidence of SARS-CoV-2 infection.

|  |  | Evidence of infection | | | | | | | | |  | | |
| --- | --- | --- | --- | --- | --- | --- | --- | --- | --- | --- | --- | --- | --- |
|  | Overall  N=10,979,467 | | Confirmed  N=608,867 | | Probable  N=776,351 | | Acute Possible  N=783,255 | | Non-acute Possible  N=51,514 | | No evidence of SARS-CoV-2  N=8,759,480 | |  |
|  | N | N | | % | N | % | N | % | N | % | N | % | p-value |
| Age |  |  | |  |  |  |  |  |  |  |  |  | <0.0001 |
| 0-17 | 2,164,998 | 79,894 | | 13.1 | 140,122 | 18.1 | 206,875 | 26.4 | 6,491 | 12.6 | 1,731,616 | 19.8 |  |
| 18-49 | 4,659,873 | 472,217 | | 77.6 | 332,122 | 42.8 | 254,317 | 32.5 | 20,831 | 40.4 | 3,580,386 | 40.9 |  |
| 50-64 | 1,573,722 | 42,667 | | 7.0 | 131,302 | 16.9 | 100,105 | 12.8 | 9,807 | 19.0 | 1,289,841 | 14.7 |  |
| 65+ | 2,580,364 | 14,089 | | 2.3 | 172,804 | 22.3 | 221,958 | 28.3 | 14,385 | 27.9 | 2,157,128 | 24.6 |  |
| Unknown/Missing | 510 | 0 | | 0.0 | 1 | 0 | 0 | 0.0 | 0 | 0.0 | 509 | 0.0 |  |
| Sex |  |  | |  |  |  |  |  |  |  |  |  | <0.0001 |
| Male | 5,703,647 | 396,692 | | 65.2 | 368,714 | 47.5 | 365,011 | 46.6 | 22,295 | 43.3 | 4,550,935 | 52.0 |  |
| Female | 5,275,685 | 212,175 | | 34.9 | 407,632 | 52.5 | 418,240 | 53.4 | 29,219 | 56.7 | 4,208,419 | 48.0 |  |
| Unknown/Missing | 135 | 0 | | 0.0 | 5 | 0 | 4 | 0 | 0 | 0.0 | 126 | 0.0 |  |
| Region of Residence |  |  | |  |  |  |  |  |  |  |  |  | <0.0001 |
| International—WHO regions |  |  | |  |  |  |  |  |  |  |  |  |  |
| African Region | 1,698 | 24 | | 0.0 | 18 | 0.0 | 12 | 0.0 | 1 | 0.0 | 1,643 | 0.0 |  |
| Region of the Americas (excluding US) | 148,740 | 24,068 | | 4.0 | 4,221 | 0.5 | 2,489 | 0.3 | 288 | 0.6 | 117,674 | 1.3 |  |
| South-East Asian Region | 1,720 | 7 | | 0.0 | 21 | 0.0 | 7 | 0.0 | 4 | 0.0 | 1,681 | 0.0 |  |
| European Region | 97,898 | 19,432 | | 3.2 | 4,149 | 0.5 | 1,731 | 0.2 | 356 | 0.7 | 72,230 | 0.8 |  |
| Eastern Mediterranean Region | 6,847 | 697 | | 0.1 | 224 | 0.0 | 103 | 0.0 | 47 | 0.1 | 5,776 | 0.1 |  |
| Western Pacific Region | 102,932 | 22,995 | | 3.8 | 2,912 | 0.4 | 1,123 | 0.1 | 152 | 0.3 | 75,750 | 0.9 |  |
| US Domestic—HHS regions |  |  | |  |  |  |  |  |  |  |  |  |  |
| Region 1 (CT, ME, MA, NH, RI, VT) | 272,832 | 3,447 | | 0.6 | 15,155 | 2.0 | 17,811 | 2.3 | 1,298 | 2.5 | 235,121 | 2.7 |  |
| Region 2 (NJ, NY) | 319,055 | 9,273 | | 1.5 | 20,138 | 2.6 | 20,594 | 2.6 | 1,606 | 3.1 | 267,444 | 3.1 |  |
| Region 3 (DE, DC, MD, PA, VA, WV) | 1,410,063 | 68,236 | | 11.2 | 93,938 | 12.1 | 93,380 | 11.9 | 6,007 | 11.7 | 1,148,502 | 13.1 |  |
| Region 4 (AL, FL, GA, KY, MS, NC, SC, TN) | 3,024,720 | 154,534 | | 25.4 | 256,962 | 33.1 | 243,470 | 31.1 | 15,692 | 30.5 | 2,354,062 | 26.9 |  |
| Region 5 (IL, IN, MI, MN, OH, WI) | 818,301 | 16,484 | | 2.7 | 58,418 | 7.5 | 62,420 | 8.0 | 4,834 | 9.4 | 676,145 | 7.7 |  |
| Region 6 (AR, LA, NM, OK, TX) | 1,634,181 | 99,333 | | 16.3 | 139,617 | 18.0 | 138,676 | 17.7 | 7,330 | 14.2 | 1,249,225 | 14.3 |  |
| Region 7 (IA, KS, MO, NE) | 457,050 | 20,715 | | 3.4 | 34,366 | 4.4 | 33,918 | 4.3 | 2,999 | 5.8 | 365,052 | 4.2 |  |
| Region 8 (CO, MT, ND, SD, UT, WY) | 534,341 | 29,046 | | 4.8 | 39,599 | 5.1 | 42,073 | 5.4 | 3,207 | 6.2 | 420,416 | 4.8 |  |
| Region 9 (AZ, CA, HI, NV) | 1,438,698 | 96,123 | | 15.8 | 75,727 | 9.8 | 83,152 | 10.6 | 5,266 | 10.2 | 1,178,430 | 13.5 |  |
| Region 10 (AK, ID, OR, WA) | 635,678 | 38,829 | | 6.4 | 29,096 | 3.8 | 41,099 | 5.3 | 2,331 | 4.5 | 524,223 | 6.0 |  |
| Unknown/Missing | 74,813 | 5,624 | | 0.9 | 1,790 | 0.2 | 1,197 | 0.2 | 96 | 0.2 | 66,106 | 0.8 |  |
| Race |  |  | |  |  |  |  |  |  |  |  |  | <0.0001 |
| American Indian or Alaska Native | 45,186 | 4,975 | | 0.8 | 3,287 | 0.4 | 2,660 | 0.3 | 219 | 0.4 | 34,045 | 0.4 |  |
| Asian or Pacific Islander | 272,112 | 31,296 | | 5.1 | 14,835 | 1.9 | 11,589 | 1.5 | 904 | 1.8 | 213,488 | 2.4 |  |
| Black or African American | 828,215 | 88,635 | | 14.6 | 48,683 | 6.3 | 42,604 | 5.4 | 2,522 | 4.9 | 645,771 | 7.4 |  |
| White | 3,451,214 | 302,091 | | 49.6 | 240,685 | 31.0 | 195,264 | 24.9 | 17,241 | 33.5 | 2,695,933 | 30.8 |  |
| Other | 204,318 | 23,503 | | 3.9 | 14,574 | 1.9 | 10,310 | 1.3 | 862 | 1.7 | 155,069 | 1.8 |  |
| Unknown/Missing | 6,178,422 | 158,367 | | 26.0 | 454,287 | 58.5 | 520,828 | 66.5 | 29,766 | 57.8 | 5,015,174 | 57.3 |  |
| Hispanic Ethnicity |  |  | |  |  |  |  |  |  |  |  |  | <0.0001 |
| Yes | 521,339 | 81,959 | | 13.5 | 31,901 | 4.1 | 23,309 | 3.0 | 1,727 | 3.4 | 382,443 | 4.4 |  |
| No | 4,278,160 | 396,053 | | 60.6 | 290,109 | 37.4 | 239,002 | 30.5 | 20,020 | 38.9 | 3,359,976 | 38.4 |  |
| Unknown/Missing | 6,179,968 | 157,855 | | 25.9 | 454,341 | 58.5 | 520,944 | 66.5 | 29,767 | 57.8 | 5,017,061 | 57.3 |  |
| MHS Beneficiary Status |  |  | |  |  |  |  |  |  |  |  |  | <0.0001 |
| Active duty^1^ | 1,889,423 | 377,152 | | 61.9 | 107,767 | 13.9 | 83,605 | 10.7 | 6,804 | 13.2 | 1,314,095 | 15.0 |  |
| Inactive Nat. Guard/Reserve | 398,318 | 9,191 | | 1.5 | 28,945 | 3.7 | 19,069 | 2.4 | 1,660 | 3.2 | 339,453 | 3.9 |  |
| Retirees | 2,360,037 | 39,054 | | 6.4 | 175,799 | 22.6 | 170,062 | 21.7 | 11,929 | 23.2 | 1,963,193 | 22.4 |  |
| Dependents | 6,206,215 | 181,546 | | 29.8 | 462,231 | 59.5 | 508,923 | 65.0 | 30,988 | 60.2 | 5,022,527 | 57.3 |  |
| Other | 124,612 | 1,923 | | 0.3 | 1,595 | 0.2 | 1,568 | 0.2 | 132 | 0.3 | 119,394 | 1.4 |  |
| Unknown | 862 | 1 | | 0.0 | 14 | 0.0 | 28 | 0.0 | 1 | 0.0 | 818 | 0.0 |  |
| Member |  |  | |  |  |  |  |  |  |  |  |  | <0.0001 |
| Self | 4,717,604 | 426,885 | | 70.1 | 313,329 | 40.4 | 273,382 | 34.9 | 20,478 | 39.8 | 3,683,530 | 42.1 |  |
| Spouse | 3,390,348 | 93,339 | | 15.3 | 283,631 | 36.5 | 267,446 | 34.2 | 22,563 | 43.8 | 2723369 | 31.1 |  |
| Child/Ward | 2,853,528 | 88,292 | | 14.5 | 179,329 | 23.1 | 242,375 | 30.9 | 8,467 | 16.4 | 2335065 | 26.7 |  |
| Parent | 17,986 | 351 | | 0.1 | 62 | 0.0 | 51 | 0.0 | 6 | 0.0 | 17516 | 0.2 |  |
| Branch of Service |  |  | |  |  |  |  |  |  |  |  |  | <0.0001 |
| Air Force | 2,878,417 | 143,209 | | 23.5 | 231,677 | 29.8 | 222,079 | 28.4 | 15,313 | 29.7 | 2,266,139 | 25.9 |  |
| Army | 4,694,753 | 278,339 | | 45.7 | 319,564 | 41.2 | 330,430 | 42.2 | 21,216 | 41.2 | 3,745,204 | 42.8 |  |
| Coast Guard | 243,516 | 5,381 | | 0.9 | 20,709 | 2.7 | 20,319 | 2.6 | 1,404 | 2.7 | 195,703 | 2.2 |  |
| Marine Corps | 862,039 | 64,343 | | 10.6 | 52,036 | 6.7 | 53,737 | 6.9 | 3,509 | 6.8 | 688,414 | 7.9 |  |
| Navy | 2,217,253 | 114,809 | | 18.9 | 149,024 | 19.2 | 153,472 | 19.6 | 9,766 | 19.0 | 1,790,182 | 20.4 |  |
| Other | 34,760 | 1,307 | | 0.2 | 495 | 0.1 | 349 | 0.0 | 88 | 0.2 | 32,521 | 0.4 |  |
| Unknown | 48,729 | 1,479 | | 0.2 | 2,846 | 0.4 | 2,869 | 0.4 | 218 | 0.4 | 41,317 | 0.5 |  |
| Rank |  |  | |  |  |  |  |  |  |  |  |  | <0.0001 |
| Enlisted E1-E3 | 794,281 | 108,210 | | 17.8 | 30,221 | 3.9 | 35,568 | 4.5 | 1,741 | 3.4 | 618,541 | 7.1 |  |
| Enlisted E4-E6 | 4,354,676 | 287,201 | | 47.2 | 311,821 | 40.2 | 316,926 | 40.5 | 17,807 | 34.6 | 3,420,921 | 39.1 |  |
| Enlisted E7-E9 | 3,223,965 | 107,783 | | 17.7 | 255,749 | 32.9 | 246,853 | 31.5 | 16,931 | 32.9 | 2,596,649 | 29.6 |  |
| Officer O1-O3 | 586,357 | 44,173 | | 7.3 | 39,299 | 5.1 | 39,762 | 5.1 | 3,254 | 6.3 | 459,869 | 5.3 |  |
| Officer O4-O6 | 1,688,988 | 47,875 | | 7.9 | 114,881 | 14.8 | 119,870 | 15.3 | 9,937 | 19.3 | 1,396,425 | 16.0 |  |
| Officer O7-O10 | 25,519 | 708 | | 0.1 | 1,591 | 0.2 | 1,663 | 0.2 | 132 | 0.3 | 21,425 | 0.2 |  |
| Warrant Officer W1-W5 | 303,963 | 12,889 | | 2.1 | 22,706 | 2.9 | 22,556 | 2.9 | 1,711 | 3.3 | 244,101 | 2.8 |  |
| Unknown | 1,718 | 28 | | 0.0 | 83 | 0.0 | 57 | 0.0 | 1 | 0.0 | 1,549 | 0.0 |  |
| Hospitalization^2^ |  |  | |  |  |  |  |  |  |  |  |  |  |
| 14 days prior to index date | 31,941 | 1,470 | | 0.2 | 5714 | 0.7 | 24,550 | 3.1 | 182 | 0.4 | -- | -- | <0.0001 |
| Index date to 30 days after | 227,410 | 10,628 | | 1.7 | 79,418 | 10.2 | 133,348 | 17.0 | 3,925 | 7.6 | -- | -- | <0.0001 |
| 31-45 days after index date | 7,093 | 1,173 | | 0.2 | 2,697 | 0.3 | 2,983 | 0.4 | 228 | 0.4 | -- | -- | <0.0001 |

^1^Active duty includes Reserve and National Guard personnel on active duty status.

^2^Beneficiaires with index dates in the last 45 days of the study period are excluded from these rows because there was not adequate follow-up. Index date used for hospitalization time frames is the day of positive laboratory test or day of medical encounter during which qualifying ICD-10-CM codes were recorded.

Abbreviations: SARS-CoV-2, severe acute respiratory syndrome coronavirus 2; WHO, World Health Organization; HHS, Health and Human Services; states use postal service abbreviations.
