# Supplementary material for: Comparing SARS-CoV-2 infections in the US Military Health System and national data: opportunities for future pandemic surveillance
Source: Front Public Health. 2026 Jan 26;13:1714024. doi: 10.3389/fpubh.2025.1714024 (PMC12883756; doi:10.3389/fpubh.2025.1714024)
Supplement: Supplementary file 4 [file Table_4.docx]

Supplementary Table 4. Spearman correlations of confirmed, probable, and acute possible SARS-CoV-2 cases in the Military Health System. Correlations are categorized as very high (0.9-1.0), high (0.7-0.89), moderate (0.5-0.69), low (0.3-0.49), and negligible (0.0-0.29).

| Case type comparison | Spearman rho | p-value |
| --- | --- | --- |
| Confirmed and probable | 0.94 | <0.0001 |
| Confirmed and acute possible | 0.50 | 0.0072 |
| Probable and acute possible | 0.73 | <0.0001 |
