# Supplementary material for: Comparing SARS-CoV-2 infections in the US Military Health System and national data: opportunities for future pandemic surveillance
Source: Front Public Health. 2026 Jan 26;13:1714024. doi: 10.3389/fpubh.2025.1714024 (PMC12883756; doi:10.3389/fpubh.2025.1714024)
Supplement: Supplementary file 5 [file Table_5.docx]

Supplementary Table 5. Spearman correlations of SARS-CoV-2 cumulative incidence between MHS beneficiaries and the general US population. Correlations are categorized as very high (0.9-1.0), high (0.7-0.89), moderate (0.5-0.69), low (0.3-0.49), and negligible (0.0-0.29). Abbreviations: HHS, Health and Human Services; MHS, Military Health System.

|  |  | Active duty Service members | | | | |  |  | Sensitivity analysis: laboratory-confirmed cases among active duty Service members | | | | |  |
| --- | --- | --- | --- | --- | --- | --- | --- | --- | --- | --- | --- | --- | --- | --- |
|  | 18-49 year olds | | | | 50-64 year olds | | | 18-49 year olds | | | 50-64 year olds | | | |
| HHS Region | Spearman rho | | | p-value | Spearman rho | p-value | | Spearman rho | | p-value | Spearman rho | p-value | | |
| Region 1 | 0.83 | | | <0.0001 | 0.64 | 0.0003 | | 0.73 | | <0.0001 | 0.67 | <0.0001 | | |
| Region 2 | 0.75 | | | <0.0001 | 0.75 | <0.0001 | | 0.71 | | <0.0001 | 0.53 | 0.0039 | | |
| Region 3 | 0.86 | | | <0.0001 | 0.87 | <0.0001 | | 0.75 | | <0.0001 | 0.80 | <0.0001 | | |
| Region 4 | 0.98 | | | <0.0001 | 0.92 | <0.0001 | | 0.96 | | <0.0001 | 0.92 | <0.0001 | | |
| Region 5 | 0.77 | | | <0.0001 | 0.83 | <0.0001 | | 0.60 | | 0.0008 | 0.71 | <0.0001 | | |
| Region 6 | 0.93 | | | <0.0001 | 0.78 | <0.0001 | | 0.87 | | <0.0001 | 0.79 | <0.0001 | | |
| Region 7 | 0.87 | | | <0.0001 | 0.62 | 0.0004 | | 0.82 | | <0.0001 | 0.76 | <0.0001 | | |
| Region 8 | 0.90 | | | <0.0001 | 0.75 | <0.0001 | | 0.89 | | <0.0001 | 0.76 | <0.0001 | | |
| Region 9 | 0.93 | | | <0.0001 | 0.82 | <0.0001 | | 0.90 | | <0.0001 | 0.93 | <0.0001 | | |
| Region 10 | 0.91 | | | <0.0001 | 0.83 | <0.0001 | | 0.88 | | <0.0001 | 0.76 | <0.0001 | | |
|  |  | | Non-active duty MHS beneficiaries | | | | | | | | | |  | |
|  | <18 year olds | | | | 18-49 year olds | | | 50-64 year olds | | | 65+ year olds | | | |
| HHS Region | Spearman rho | | | p-value | Spearman rho | p-value | | Spearman rho | | p-value | Spearman rho | p-value | | |
| Region 1 | 0.81 | | | <0.0001 | 0.82 | <0.0001 | | 0.79 | | <0.0001 | 0.67 | 0.0001 | | |
| Region 2 | 0.75 | | | <0.0001 | 0.71 | <0.0001 | | 0.69 | | <0.0001 | 0.48 | 0.01 | | |
| Region 3 | 0.86 | | | <0.0001 | 0.88 | <0.0001 | | 0.90 | | <0.0001 | 0.65 | 0.0002 | | |
| Region 4 | 0.87 | | | <0.0001 | 0.91 | <0.0001 | | 0.86 | | <0.0001 | 0.72 | <0.0001 | | |
| Region 5 | 0.92 | | | <0.0001 | 0.86 | <0.0001 | | 0.84 | | <0.0001 | 0.67 | <0.0001 | | |
| Region 6 | 0.82 | | | <0.0001 | 0.89 | <0.0001 | | 0.89 | | <0.0001 | 0.77 | <0.0001 | | |
| Region 7 | 0.92 | | | <0.0001 | 0.86 | <0.0001 | | 0.85 | | <0.0001 | 0.73 | <0.0001 | | |
| Region 8 | 0.91 | | | <0.0001 | 0.87 | <0.0001 | | 0.85 | | <0.0001 | 0.68 | <0.0001 | | |
| Region 9 | 0.80 | | | <0.0001 | 0.85 | <0.0001 | | 0.85 | | <0.0001 | 0.57 | 0.0014 | | |
| Region 10 | 0.91 | | | <0.0001 | 0.87 | <0.0001 | | 0.89 | | <0.0001 | 0.60 | 0.0008 | | |
