# Supplementary figures and images for: Comparing SARS-CoV-2 infections in the US Military Health System and national data: opportunities for future pandemic surveillance
Source: Front Public Health. 2026 Jan 26;13:1714024. doi: 10.3389/fpubh.2025.1714024 (PMC12883756; doi:10.3389/fpubh.2025.1714024)

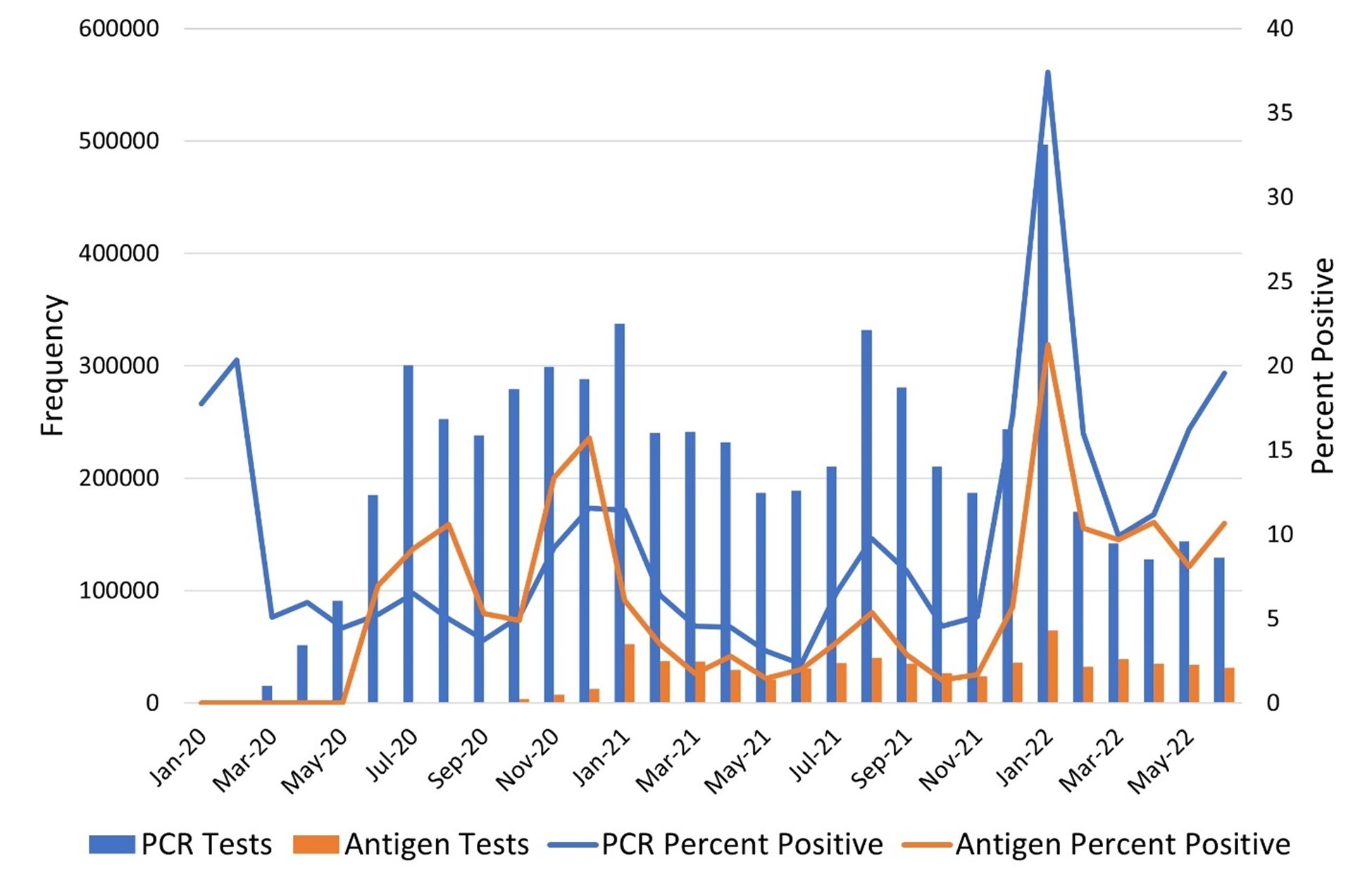

Supplement: Supplementary file 6 [file Image_1.jpg]

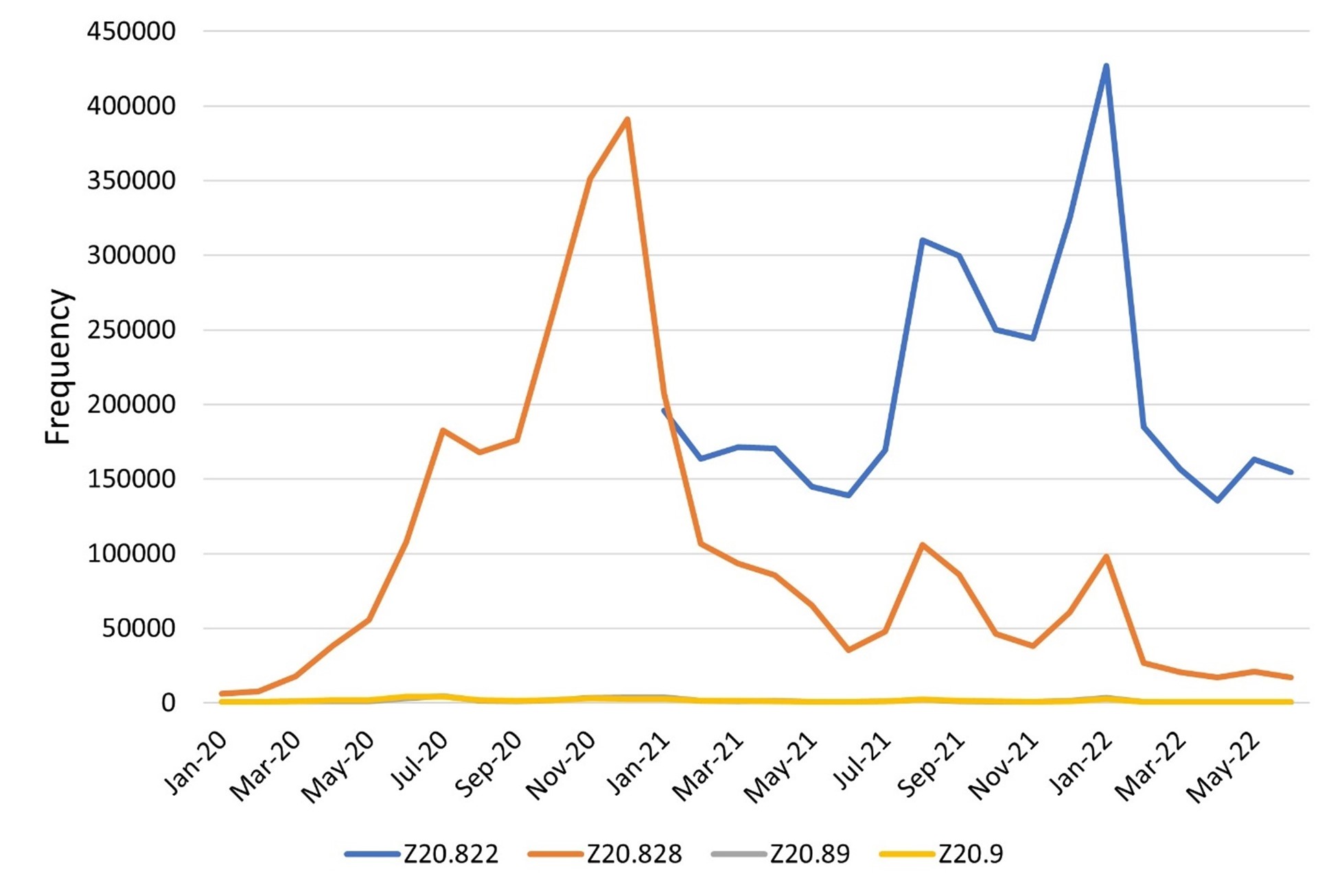

Supplement: Supplementary file 7 [file Image_2.jpg]
